# Correlation between vessel density and thickness in the retina and choroid of severe non-proliferative diabetic retinopathy patients

**DOI:** 10.3389/fendo.2024.1373363

**Published:** 2024-05-14

**Authors:** Kai He, Selena Wei-Zhang, Ziqi Li, Parhat Kaysar, Tianjing Yang, Zhiyong Sun, Wei Zhou, Hua Yan

**Affiliations:** ^1^ Department of Ophthalmology, Tianjin Medical University General Hospital, Tianjin, China; ^2^ School of Medicine, Nankai University, Tianjin, China; ^3^ Ministry of Education International Joint Laboratory of Ocular Diseases, Tianjin, China; ^4^ Tianjin Key Laboratory of Ocular Trauma, Tianjin, China; ^5^ Tianjin Institute of Eye Health and Eye Diseases, Tianjin, China; ^6^ China-UK “Belt and Road” Ophthalmology Joint Laboratory, Tianjin, China

**Keywords:** retinal thickness, optical coherence tomography angiography, severe nonproliferative diabetic retinopathy, vessel density, choroidal thickness

## Abstract

**Objectives:**

To explore the correlation between the vessel density (VD) of the retina and choroid vascular plexuses and the thicknesses of their respective retinal layers and choroid membranes in participants with severe non-proliferative diabetic retinopathy (NPDR).

**Methods:**

We retrospectively analyzed the data of 42 eyes of 42 participants with diabetes mellitus (DM) and severe NPDR. In addition, 41 eyes of 41 healthy controls were evaluated. Measurements were taken for both groups using optical coherence tomography angiography (OCTA), including the area and perimeter of the foveal vascular zone (FAZ) and the vascular density (VD) in the superficial capillary plexus (SCP), deep capillary plexus (DCP), and choroid capillary (CC). These measurements were compared with the retinal thickness (RT) of the inner/intermediate retinal layers and choroidal thickness (CT). The study evaluated the correlation between RT or CT and VD in the respective vascular networks, namely superficial capillary plexus (SCP), deep capillary plexus (DCP), or CC.

**Results:**

The inner RT and VD in all plexuses were significantly lower in the severe NPDR group than in the healthy controls. Furthermore, the FAZ area and perimeter were larger in the severe NPDR group. Inner RT was correlated with VD in the SCP group (r=0.67 and r=0.71 in the healthy control and severe NPDR groups, respectively; p<0.05). CT negatively correlated with VD in the CC (r=-0.697 and r=-0.759 in the healthy control and severe NPDR groups, respectively; p<0.05). Intermediate RT significantly correlated with VD in the DCP of the severe NPDR group (r=-0.55, p<0.05), but not in the healthy control group.

**Conclusions:**

Retinal or choroidal thickness strongly correlated with VD. Therefore, patients with severe NPDR must consider the distinct anatomical and functional entities of the various retinal layers and the choroid.

## 1 Introduction

Diabetic retinopathy (DR) is a prevalent complication of DM, particularly in the working-age population, representing a primary cause of blindness (1). Severe NPDR is the severe pathological stage of diabetic retinopathy at which DR is most commonly recommended for clinical treatment (2). Fluorescent angiography and fundus photography have been used to diagnose DR. OCTA is a form of non-invasive examination that can provide elaborate images without dye injection. It is increasingly used for diabetic retinopathy diagnosis and observational studies owing to its high resolution and noninvasiveness.

DR leads to structural and functional changes in the retina, microaneurysm formation, non-perfusion areas, and vascular abnormalities, which are the main pathological features of severe NPDR (3). In this context, OCTA can acquire more information, allowing quantitative analysis of the choroid and retina. The majority of the literature has demonstrated the potential role of OCTA in DR, such as in examining neovascular complexes (4) and non-perfusion areas (5). VD, as well as the FAZ area in the retina of diabetic patients are altered compared with those of healthy people (6, 7). Moreover, the structure of retinal changes, even in diabetic patients who do not have DR (8, 9), included a thinner retinal sublayer, including the ganglion cell layer (GCL), retinal nerve fiber layer (RNFL), and inner plexiform layer (IPL).

Several studies have previously investigated on the correlation between the retinal structure and VD, providing particular insights into diagnosing and treating fundus diseases (10, 11). Some researchers (12) have further demonstrated that the OCTA parameters for the VD and FAZ circularity indices were correlated with the GCL/IPL in diabetic patients with diabetes. Decreased choroidal VD can lead to structural changes in the retina (13). Choroidal VD has been suggested to correlate with retinal thickness. However, relevant studies investigating the correlation between retinal/choroidal thickness and OCTA-related parameters need to be performed, particularly in patients with severe NPDR.

In this study, we focused on identifying changes in VD and retinal sublayer/choroidal thickness, as well as identifying the correlation between thickness and VD in patients with severe NPDR.

## 2 Materials and method

### 2.1 Participants

Participants with diabetes who underwent DR-related fundus examinations were admitted to Tianjin Medical University General Hospital between March 2021 and March 2023. Age-matched healthy controls were included in this cross-sectional study. Two ophthalmologists evaluated and graded the fundus images. All participants who were diagnosed as severe NPDR without the presence of diabetic macular edema (DME) were enrolled in this research. Participants who had undergone fundus therapy, such as anti-VEGF therapy and/or laser therapy, or had any other conditions that could affect the microvessels of the choroid and retina, such as retinal vascular obstruction, glaucoma, or proportional retinal disease, were excluded.

Relevant demographic data and clinical information, including age, sex, axial length, body mass index (BMI), smoking status, blood pressure, best-corrected visual acuity (BCVA), and intraocular pressure (IOP), were collected.

This study was conducted in accordance with the principles of the Declaration of Helsinki, and was approved by the Ethics Committee of the Tianjin Medical University General Hospital (approval number (RB2021-YX-048–01). Consent was obtained from the participants, all of whom were made aware of the purpose and potential outcomes of the study and willingly agreed to participate. The participants in this project were well informed about the study’s objectives and potential effects, and they willingly provided their consent.

### 2.2 Swept-source optical coherence tomography and OCTA imaging and measurements

Images derived by Swept-source optical coherence tomography and OCTA were captured by a Zeiss Cirrus (HD-OCT 5000) equipped with an Angioplex (Carl Zeiss Meditec, Dublin, CA, USA). A 3x3 mm scan was created for each eye, focusing on the central part of the foveal area. FastTrac retinal tracking technology (San Francisco, CA, USA) was used to minimize motion artifacts. A signal strength > 6 out of 10 was accepted. En-face OCTA images were automatically generated using an optical microangiography algorithm of the Angioplex software. The SCP, DCP, and CC were automatically identified using a review software program (Carl Zeiss Meditec). The SCP and DCP were defined as the inner limiting membrane to the inner plexiform layer and from the inner nuclear layer to the outer plexiform layer, respectively (14). CCP was defined as 10 µm thickness under the complex of retinal pigment epithelium and Bruch membrane (15). The thickness of the central macula was measured manually by an examiner who was unaware of the details. We further determined choroid thickness as the length from the outer border of the retinal pigment epithelium to the sclero-choroidal interface. The thickness of the inner retinal layer was defined from the inner membrane layer to the IPL, whereas that for the intermediate retinal layer was defined from the inner nuclear layer (INL) to the outer plexiform layer (OPL).

### 2.3 Quantitative OCTA image analysis

As previously mentioned, the binarization processing of the SCP and DCP was conducted using customized Python 3.5 code, provided by The Python Software Foundation (United States) (16). In summary, each image was subjected to a top-hat filter, followed by further processing. Two distinct techniques for binarization were employed: the initial image was processed with a Hessian filter, and subsequently subjected to global thresholding using Huang’s fuzzy thresholding approach. A median local thresholding approach was applied to the second image. Combining the two processed images results in the creation of an ultimate binarized image. The foveal circle was surrounded by an annular region. Pixels in both images were exclusively considered when included in the analysis. For the analysis, a combination of automated outlining using review software and manual outlining by two separate investigators was utilized, with manual outlining employed in cases where the algorithm signals were not strong. The diameter of the CC OCTA image was binarized using the Phansalkar method to calculate the VD, which is represented as the percentage of the total vessel area divided by the total measured area (1–3 mm in diameter) (8), as mentioned earlier (15).

### 2.4 Statistical analysis

The statistical analyses utilized IBM SPSS Statistics for Windows (version 25.0). Categorical variables are described as numbers or percentages. Pearson’s chi-square test was conducted on both the healthy control and severe NPDR groups, with continuous variables presented as the mean and standard deviation (SD). Student’s *t*-test was conducted to compare two groups. We used a single-factor regression analysis to investigate the correlation between VD and VD in both groups. Statistical significance was set at p < 0.05.

## 3 Results

**Table 1** summarizes the demographic characteristics of the 83 participants involved in the study. Overall, we examined 41 eyes from 41 healthy controls (age: mean (SD), 54.6 (12.5) years) and 42 eyes from 42 participants with severe NPDR (age: mean (SD), 52.3 (8.7) years). No notable disparities were noted in terms of sex, axial length, BMI, smoking status, blood pressure, or IOP (p>0.05), although the BCVA of the healthy control group (logMAR 0.02 (0.05)) was better than that of the severe NPDR group (LogMAR 0.26 (0.25)) (p<0.05).

**Table 1:** Basic information of healthy controls and severe NPDR patients.

Characteristic Means ± SD/n (%)	Healthy controls (n=41)	Severe NPDR patients (n=42)	p value
Age, year	54.6 ± 12.5	52.3 ± 8.7	0.318
BMI, kg/m^2^	25.6 ± 3.1	24.7 ± 3.0	0.202
Blood pressure, mmHg	143.7 ± 17.8	141.7 ± 18.9	0.619
Axial length, mm	23.7 ± 1.4	23.5 ± 1.2	0.446
BCVA, logMAR	0.02 ± 0.05	0.26 ± 0.25	<0.001
IOP, mmHg	16.2 ± 3.1	17.4 ± 2.5	0.061
Sex (male)	24 (58.5)	24 (57.1)	0.89
Smoking (yes)	16 (39.0)	16 (38.1)	0.93

BMI, body mass index; BCVA, best-corrected visual acuity; IOP, intraocular pressure; SD, standard deviation; NPDR, nonproliferative diabetic retinopathy.

**Table 2** shows the comparisons between the thickness of the retinal layers (CMT and sublayers) and choroid thickness. In the severe NPDR group, the inner retinal thickness was significantly thinner than that in the healthy control group (107.10 (4.96) vs. 116.0 (4.99), respectively, p<0.05); conversely, the severe NPDR group had a thicker CMT than the healthy control group (277.4 (73.6) vs. 253.5 (18.9)). Furthermore, no significant difference was observed in the intermediate retinal thickness between the two groups (72.00 (10.05) vs. 71.44 (6.18), p>0.05). The severe NPDR group had a thinner choroid than the healthy control group (429.9 (65.7) vs. 464.5 (71.0)).

**Table 2:** Results of parafoveal VD, FAZ, parafoveal RT, and CT in the two groups.

Characteristic Means ± SD		Healthy control (n=41)	Severe NPDR patients (n=42)	p value
VD	SCP	30.23 ± 2.72	28.05 ± 3.74	0.003
	DCP	29.30 ± 1.70	26.81 ± 2.57	<0.001
	CC	64.20 ± 2.40	61.7 ± 4.2	0.001
FAZ	Area, µm^2^	0.37 ± 0.10	0.56 ± 0.22	<0.001
	Perimeter, µm	2.27 ± 0.30	2.84 ± 0.61	<0.001
	Acircularity index	1.59 ± 0.03	1.10 ± 0.07	0.016
RT	ILM-IPL	116.0 ± 4.99	107.10 4.96	<0.001
	INL-OPL	71.44 ± 6.18	72.00 10.05	0.761
	Central macular thickness	253.50 ± 18.90	277.40 ± 73.60	0.049
CT		464.50 ± 71.00	429.9 ± 65.70	0.024

Values are expressed as mean ± SD.

CC, choroid capillary; CT, choroidal thickness; VD, vessel density; NPDR, non-proliferative diabetic retinopathy; FAZ, foveal vascular zone; RT, retinal thickness; SCP, superficial capillary plexus; DCP, deep capillary plexus; IPL, inner plexiform layer; ILM, internal limiting membrane; INL, inner nuclear layer; OPL, outer nuclear layer; SD, standard deviation.

**Figure 1:**
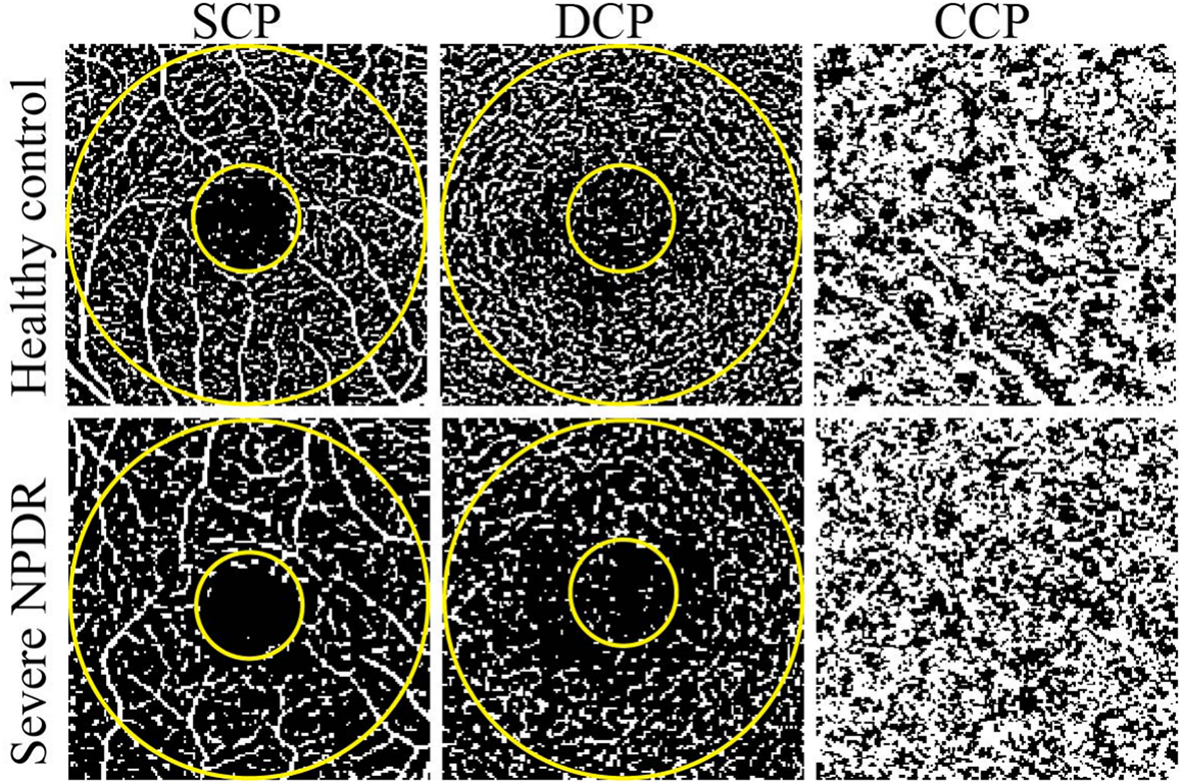
Representative OCTA images of healthy control and severe NPDR patients. The left, middle, and right represent OCTA images of the SCP, DCP, and CCP, respectively. The evaluated regions consisted of a circular area with a diameter of 1 mm at the center of the fovea and a donut-shaped area surrounding it, with diameters ranging from 1 to 3 mm.

We further compared the VD and FAZ parameters between the healthy control and severe NPDR groups (**Table 2**; **Figure 1**). Overall, the parafoveal VD of SCP and DCP in the severe NPDR group were significantly lower than that in the healthy control group ((28.05 (3.74) vs. 30.23 (2.72), p<0.05, and 26.81 (2.57) vs. 29.30 (1.70), p<0.05, respectively). Additionally, the severe NPDR group had a larger FAZ area and perimeter than the healthy control group (0.56 (0.22) vs. 0.37 (0.10), p<0.05, and 2.84 (0.61) vs. 2.27 (0.30), p<0.05, respectively). The acircularity index of the FAZ was 1.10 (0.07) in the severe NPDR group and 1.59 (0.03) in the healthy control group, with significant difference between the groups (p<0.05).

**Figure 2:**
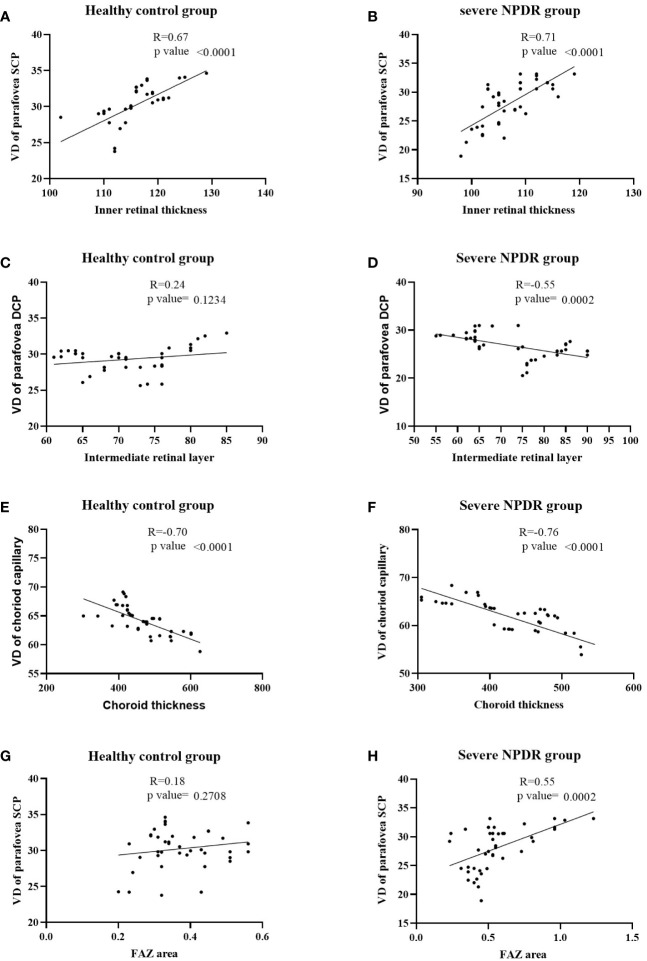
Correlations between vessel density and capillary plexuses. **(A, B)** Correlation between inner retinal thickness and VD of the parafoveal SCP in the healthy control and severe NPDR groups; **(C, D)** correlation between intermediate retinal thickness and VD of the parafoveal DCP in the healthy control and severe NPDR groups; **(E, F)** correlation between choroid thickness and VD of choroid capillaries in the healthy control and severe NPDR groups; **(G, H)** correlation between FAZ area and VD of the parafoveal SCP in the healthy control and severe NPDR groups. VD, vessel density; NPDR, non-proliferative diabetic retinopathy; FAZ, foveal vascular zone; SCP, superficial capillary plexus; DCP, deep capillary plexus.

A strong connection was found in the vascular density of the parafoveal SCP and the thickness of the inner retina in the healthy control group (r=0.67, p<0.001; **Figure 2A**) and severe NPDR groups (r=0.71, p<0.001; **Figure 2B**). However, the VD of the parafoveal DCP was not significantly associated with intermediate retinal thickness (p>0.05; **Figures 2C, D**). Analysis of the correlation between choroidal thickness and capillary VD demonstrated negative correlations in both severe NPDR (r=-0.70, p<0.001; **Figure 2E**) and healthy control groups (r=-0.76, p<0.001; **Figure 2F**). Interestingly, we also found a positive correlation between the FAZ area and the SCP of the VD in the severe NPDR group (r=0.55, p<0.000; **Figure 2G**), but not in the healthy control group (r=0.18, p>0.05; **Figure 2H**).

## 4 Discussion

In this study, we examined variations in retinal or choroidal thickness and VD in participants with severe NPDR. We further explored the connection between VD in the retinal plexus or choriocapillaris and the corresponding thickness in the eyes of healthy participants and those with severe NPDR but without DME.

The effect of DM on retinal thickness has been validated in many studies, with results showing that DM affects the RNFL, GCL, and IPL in diabetic patients with (8, 9) or without DR (17). Our results showed that the inner retinal thickness decreased in the severe NPDR group. Kim et al. (18) reported that progressive damage to the mGCIPL affected the progression of patients with early stage DR. Simultaneously, researchers found that patients with DR had lower inner retinal thickness than controls (19). DR is an ischemic disease simultaneously affecting all retinal layers (10, 11). The central retinal vasculature supplied the inner retinal layer. DR causes microvascular dysfunction, leading to chronic retinal ischemia. Structural damage to the inner retina is also considered an essential manifestation of DR, as DR causes damage or apoptosis in many cell types in the inner retina (20).

Several studies have reported larger FAZ areas and perimeters in patients with DM (6, 21). OCTA has allowed the capture of detailed images of patients with DM, enabling physicians to make advanced decisions and analysis. Changes in FAZ can be observed in patients with DM, although they do not show signs of DR (6). In our study, the severe NPDR group showed a tendency toward an expanded FAZ area compared to the healthy control group, which has been verified in many studies (6, 7). Although changes in the FAZ area have been treated as a vital fundus indicator of early DR, they are also considered an essential parameter for measuring DR severity (22). Bresnick et al. (23) reported that FAZ enlargement is caused by capillary loss in the adjacent vessels. Moreover, the severity of microvascular changes and macular ischemia increases with DR progression (24). In addition, we found a positive correlation between the FAZ area and perimeter and retinal VD in the severe NPDR group, but not in the healthy control group. The enlargement of the FAZ area and perimeter is a manifestation of retinal hypoxia. Compared with other DR-related parameters such as VD, spacing between vessels, and perfusion density, the FAZ area showed lower sensitivity and specificity (21). Based on our results, we believe that changes in the FAZ area, combined with other indicators such as VD, can be used to indicate DR severity.

Several studies have previously demonstrated a correlation between choroidal angiopathy and retinopathy in patients with DR (25, 26). Although indocyanine green angiography can be used to detect choroidal structure and function, OCTA provides real-time imaging with precise anatomical details and quantification of the choroid *in vivo*. In our study, patients with severe NPDR exhibited a thinner choroid than healthy controls and a decrease in CC was observed in the severe NPDR group. Mehreen et al. (27) reported a reduction in the CC of eyes with PDR and DME compared to controls. Regatieri et al. (25) also found that choroidal thickness is altered in diabetes, and may be related to the severity of retinopathy. Choi et al. (13) further found that microvascular abnormalities of the CC occurred in all DR stages. These findings are consistent with our results. In addition, our results showed that both healthy controls and patients with severe NPDR showed a negative correlation between the VD of the CC and choroidal thickness. In support of this, Schocket et al. (28) and Lutty et al. (29) found that the choriocapillaris in eyes with moderate-to-severe DR was closed, which could be the reason for the decrease in the VD of the CC and choroidal thickness. In our study, although only the entire thickness of the choroid and VD of the CC were measured, our findings of decreased choroidal thickness and VD of the CC are consistent with those of Mehreen et al. (27). Although the CC accounts for only 5–10% of the choroid membrane the CC layer accounts for (30), this layer of blood vessels may be involved in the pathological process of DR.

## 5 Limitations

In this project, we included only a limited number of eyes; thus, the study has limited power. Moreover, this was a retrospective study, and we did not obtain fundus data from patients with severe NPDR before DR occurred.

## 6 Conclusion

Our study demonstrated a decreasing tendency in retinal or choroidal thickness and VD in patients with severe NPDR, and further showed a correlation between VD and the corresponding thickness. DR affects both the retina and choroid. Our study elucidated the pathological characteristics of the ocular structure in patients with severe NPDR. Numerous OCTA parameters suggest an intrinsic correlation that may provide a new strategy for the pathogenesis and diagnosis of DR.

## Data availability statement

The raw data supporting the conclusions of this article will be made available by the authors, without undue reservation.

## Ethics statement

The studies involving humans were approved by the ethics committee at Tianjin Medical University. The studies were conducted in accordance with the local legislation and institutional requirements. The participants provided their written informed consent to participate in this study. Written informed consent was obtained from the individual(s) for the publication of any potentially identifiable images or data included in this article.

## Author contributions

HY: Funding acquisition, Supervision, Validation, Writing – review & editing. KH: Data curation, Formal analysis, Investigation, Software, Writing – original draft. SW-Z: Data curation, Investigation, Software, Writing – original draft. ZL: Data curation, Investigation, Writing – original draft. PK: Data curation, Investigation, Writing – original draft. TY: Writing – original draft. ZS: Data curation, Investigation, Writing – original draft. WZ: Investigation, Supervision, Validation, Writing – review & editing.
